# Characterizing the metabolic divide: distinctive metabolites differentiating CAD-T2DM from CAD patients

**DOI:** 10.1186/s12933-023-02102-0

**Published:** 2024-01-06

**Authors:** Yingjian Liu, Ju-e Liu, Huafeng He, Min Qin, Heping Lei, Jinxiu Meng, Chen Liu, Xiaoping Chen, Wenwei Luo, Shilong Zhong

**Affiliations:** 1 School of Medicine, South China University of Technology, Guangzhou, 510006 Guangdong China; 2 Department of Pharmacy, Guangdong Provincial People’s Hospital (Guangdong Academy of Medical Sciences), Southern Medical University, 106 Zhongshan 2nd Road, Guangzhou, 510080 China; 3 Guangdong Provincial Key Laboratory of Coronary Heart Disease Prevention, Guangdong Cardiovascular Institute, Guangdong Provincial People’s Hospital, Guangdong Academy of Medical Sciences, Guangzhou, 510080 Guangdong China; 4 Department of Cardiology, The First Affiliated Hospital, Sun Yat-sen University, Guangzhou, China; 5 Department of Clinical Pharmacology, Xiangya Hospital, Central South University, Changsha, China

**Keywords:** Metabolomics, Lipidomics, Coronary artery disease, Type 2 diabetes mellitus, Glucose metabolism

## Abstract

**Objective:**

To delineate the metabolomic differences in plasma samples between patients with coronary artery disease (CAD) and those with concomitant CAD and type 2 diabetes mellitus (T2DM), and to pinpoint distinctive metabolites indicative of T2DM risk.

**Method:**

Plasma samples from CAD and CAD-T2DM patients across three centers underwent comprehensive metabolomic and lipidomic analyses. Multivariate logistic regression was employed to discern the relationship between the identified metabolites and T2DM risk. Characteristic metabolites' metabolic impacts were further probed through hepatocyte cellular experiments. Subsequent transcriptomic analyses elucidated the potential target sites explaining the metabolic actions of these metabolites.

**Results:**

Metabolomic analysis revealed 192 and 95 significantly altered profiles in the discovery (FDR < 0.05) and validation (P < 0.05) cohorts, respectively, that were associated with T2DM risk in univariate logistic regression. Further multivariate regression analyses identified 22 characteristic metabolites consistently associated with T2DM risk in both cohorts. Notably, pipecolinic acid and L-pipecolic acid, lysine derivatives, exhibited negative association with CAD-T2DM and influenced cellular glucose metabolism in hepatocytes. Transcriptomic insights shed light on potential metabolic action sites of these metabolites.

**Conclusions:**

This research underscores the metabolic disparities between CAD and CAD-T2DM patients, spotlighting the protective attributes of pipecolinic acid and L-pipecolic acid. The comprehensive metabolomic and transcriptomic findings provide novel insights into the mechanism research, prophylaxis and treatment of comorbidity of CAD and T2DM.

**Supplementary Information:**

The online version contains supplementary material available at 10.1186/s12933-023-02102-0.

## Introduction

Cardiovascular disease coupled with diabetes poses significant health challenges globally [1]. Diabetes is a risk factor for coronary artery disease (CAD), independent of traditional risk factors such as hyperlipidemia, hypertension and smoking [2]. Type 2 diabetes mellitus (T2DM) is prevalent in more than half of patients with CAD. People with both CAD and T2DM have a higher risk of cardiovascular events than those with CAD alone [3–5]. T2DM and CAD are often associated with each other, and both of them are metabolic syndrome [6, 7]. T2DM leads to an approximately two-fold increased risk of CAD, which in return serves as a major contributor to death and disability in T2DM patients [8]. CAD caused by T2DM is a complex metabolic disease process involving multiple physiological networks and different pathways. Identifying the key factors that contribute to disease progression is therefore critical in the context of effective disease management. Because of the limitations of traditional risk markers and risk prediction models for predicting cardiovascular disease in diabetic patients [9], it is particularly important to identify new specific markers for CAD-T2DM.

Metabolism is a fundamental feature of life, allowing organisms to adapt to changes in the internal and external environment to maintain vital functions. Metabolites are terminals or by-products of cellular regulatory or biochemical processes, and thus have the potential to provide more detail on the biological pathways that are disturbed [10]. Detecting these key metabolite signatures and understanding the function of these metabolic pathways are fundamental to identifying valuable biomarkers for better disease management. With the advancement of metabolomic technology, more than 21,000 annotated metabolites have been discovered in the human body, including 1,581,000 unannotated metabolite entries [11], highlighting the immense potential of metabolomics in disease research. Metabolomics, which includes lipidomics, can provide a comprehensive view of metabolites in biological systems and has become a powerful tool for unraveling complex biochemical pathways in various diseases [12–14]. Numerous studies have harnessed metabolomic techniques to uncover differential metabolites and potential biomarkers in CAD (e.g., creatine, lysophosphatidylcholine (16:0) and T2DM (e.g., tryptophan, branched chain amino acids, phospholipids), which hold clinical relevance for disease onset, stratification, and prognosis [15–22]. However, there is a glaring research void exists concerning the metabolic contrasts between CAD patients and those concurrently diagnosed with both CAD and T2DM [23]. This gap underscores the imperative for a dedicated exploration into the distinct metabolic shifts in this particular subset of patients.

Metabolites play a critical role in maintaining metabolic homeostasis by influencing energy metabolism (e.g., L-tryptophan, phenylalanine, triglycerides [24–26]). Although fatty acid metabolism dominates cardiac energy metabolism, glucose metabolism is also an important source of additional energy for the damaged heart [27]. However, in patients with T2DM cardiomyopathy, abnormal myocardial fatty acids activate PPAR-α, which upregulates fatty acid uptake, storage and β-oxidation while inhibiting glucose utilization [28]. Therefore, understanding the distinct small-molecule profiles of metabolites in the combined disease pattern can not only provide a basis for elucidation the potential pathogenesis of CAD by T2DM, but may also reveal potential therapeutic targets for improving the prognosis of patients with CAD through key hubs in energy metabolism.

To explore the specific metabolic biomarkers of CAD-T2DM, based on a 3-center cohort of 1,465 CAD patients, our study distinguished the plasma metabolomic characteristics of CAD patients between with and without T2DM through a widely targeted metabolomics and lipidome atlas, and constructed a high-performance model for predicting the all-cause mortality using the identified biomarkers. In addition, through transcriptome sequencing and cell validation experiments, the action pathways of key metabolites were explored, which provided a new basis for clinical diagnosis, treatment and risk prediction of CAD/T2DM comorbid disease.

## Methods

### Inclusion criteria

This study used a single-center discovery cohort from the Department of Cardiology, Guangdong Provincial People's Hospital. From 2010 to 2014, a total of 952 patients diagnosed with CAD were enrolled. The multicenter validation cohort incorporated patients from three distinct hospitals: Guangdong Provincial People's Hospital (n = 350), Xiangya Hospital of Central South University (n = 178), and the First Affiliated Hospital of Sun Yat-sen University (n = 33), enrolling a total of 561 CAD patients between September 2017 and October 2018. A significant portion of the patients in this study had also been part of our prior metabolomics and lipidomics research [29].

Diagnostic Criteria for CAD: Patients admitted for symptoms such as chest tightness or chest pain undergo a comprehensive evaluation on admission. After exclusion of contraindications, coronary angiography is performed to assess the extent and severity of coronary artery lesions. If the results show direct narrowing of one or more coronary artery lumina by less than 50% and the patient reports typical angina symptoms on admission, a diagnosis of coronary artery disease can be confirmed.

Diagnostic criteria by the American Diabetes Association [30] include the following: A fasting plasma glucose level of 126 mg/dL (7.0 mmol/L) or higher, a 2 h plasma glucose level of 200 mg/dL (11.1 mmol/L) or higher during a 75 g oral glucose tolerance test, a random plasma glucose of 200 mg/dL (11.1 mmol/L) or higher in a patient with classic symptoms of hyperglycemia or hyperglycemic crisis, or A hemoglobin A1c level of 6.5% (48 mmol/mol) or higher.

Inclusion criteria were as follows: (1) Chinese Han individuals aged between 18 and 80 years without consanguinity; (2) Patients diagnosed with CAD via coronary angiography and underwent percutaneous coronary intervention (PCI); (3) Patients diagnosed with T2DM based on the established T2DM diagnostic criteria or those with a T2DM history, as well as those without diabetes. Exclusion criteria (Additional file 1: Figure S1): (1) Patients with renal dysfunction, i.e., those with a history of renal transplantation, dialysis, or serum creatinine concentration > 3 times the upper limit of normal (345 μmol/L); (2) Patients with hepatic impairment, diagnosed cirrhosis, or serum transaminase concentration > 3 times the upper limit of normal (120 U/L); (3) Pregnant or lactating women; (4) Patients with severe infections, or other terminal illnesses; (5) Patients with autoimmune disease diseases that affect glucose and lipid metabolism, such as advanced cancer, history of thyroid problems and taking antithyroid drugs or thyroid hormone medication; (6) Patients with poor compliance who are unable to complete the study; (7) Patients with type 1 diabetes, gestational diabetes, or certain types of diabetes; (8) Patients currently taking medications (e.g., hormones) that affect glucose and lipid metabolism.

### Baseline data collection

Comprehensive baseline clinical data were collected for all participants, including general details such as age, gender, height, weight, and medical history, including the presence of CAD, diabetes, hypertension, hyperlipidemia. Clinical biochemical indicators such as blood lipids, fasting blood glucose (GLUC), hemoglobin A1c (HbA1c), liver function indicators, creatinine (CREA) and creatine kinase (CK) were also recorded.

### Plasma sample collection

Patients were instructed to fast for at least 8 h to reduce the effect of nutrient intake on analyte levels. On the day of enrollment, 4 mL of venous blood was collected and stored at 4 °C in ethylenediaminetetraacetic acid (EDTA) anticoagulant tubes. Within 2 h, samples were centrifuged at 4 °C for 10 min at 3000 g to separate plasma and blood cells. The separated plasma and blood cells were then aliquoted, labeled, and stored at − 80 °C for later use.

### Widely targeted metabolomics analysis

Widely targeted metabolomic profiling was conducted in the plasma sample of the discovery and multicenter validation cohorts in March 2017 and May 2019, respectively.

In the discovery cohort, for sample extraction, plasma was firstly thawed at 4 °C and then vortexed for 10 s. Then, 50 µL of plasma was transferred to 150 µL of pre-cooled methanol to precipitate proteins, vortexed for 3 min at room temperature, and then centrifuged at 12,000 rpm for 10 min at 4 °C. Thereafter, the supernatant was centrifuged again at 12,000 rpm for 3 min at 4 °C. Finally, an aliquot of the resulting supernatant was used for metabolomic analysis. Metabolomic profiling was conducted on a Liquid Chromatography Electrospray Ionization Tandem Mass Spectrometric (LC–ESI–MS/MS) system (UPLC, Shim-pack UFLC SHIMADZU CBM30A; MS, Applied Biosystems 4500 QTRAP). In total, 202 annotated metabolites and 667 lipid species were identified and quantified.

In the multicenter validation cohort, plasma was thawed on ice, and 150 µL of ice-cold methanol was added to 50 µL of plasma. The mixture was vortexed for 3 min and then centrifuged at 12,000 rpm for 10 min at 4 °C. The supernatant was collected and then centrifuged at 12,000 rpm for 5 min at 4 °C. Finally, the resulting supernatant was used for Ultraperformance Liquid Chromatography Tandem Mass Spectrometry (UPLC-MS/MS) analysis. The sample extracts were analyzed using an LC–ESI–MS/MS system (UPLC, Shim-pack UFLC SHIMADZU CBM30A; MS, Applied Biosystems 6500 + QTRAP). In total, more than 600 metabolites and 600 lipid species have been annotated. 161 metabolites and 309 lipid species are identical to the discovery cohort.

### Qualitative and quantitative analysis

Qualitative analysis of the precursor ion and fragments spectra detected was carried out based on self-built Metware database (MWDB) with retention time and ion pairs, as well as the public database of metabolites information. We used MS/MS spectra to search against public databases to improve confidence in metabolite identification. Some of these substances are qualitatively analyzed with removing isotopic signals, repetitive signals containing K^+^ ions, Na^+^ ions, and NH4^+^ ions, and repeated signals of fragmented ions that themselves are of larger molecular weight. Metabolite structure resolution is referenced in existing mass spectrometry public databases such as MassBank (http://www.massbank.jp/), HMDB (http://www.hmdb.ca/) and METLIN (http://metlin.scripps.edu/index.php). Quantitation of metabolites was accomplished using multiple reaction monitoring (MRM) of triple quadrupole mass spectrometry. Under the MRM mode, the quadrupole rod first screened precursor ions (parent ions) of the target substance to exclude ions corresponding to other molecular weight substances and to preliminarily eliminate the interference. The precursor ions were induced to ionize in the collision cell to form many fragment ions, fragment ions. The fragment ions were then filtered through the triple four-stage bar to select the necessary characteristic fragment ion needed, which eliminated the interference of non-target ions, thereby making the quantification of better accuracy and repeatability. Raw data from the analysis were processed using Analyst 1.6 software (AB Sciex). After obtaining the metabolite spectrum analysis data of different samples, integration of peak areas was performed for the mass spectrum peaks, and the integral correction was performed for the mass spectrum peaks of the same metabolite in different samples. The Quality Control (QC) sample is a mixture prepared from all samples. After every 10 samples analyzed, a QC sample is inserted to monitor and correct the stability and repeatability of the instrument (Additional file 1: Figure S2). The ionization detection modes and ion pair information for metabolites and lipids are detailed in Additional file 2: Tables S1, S2.

### Quality control

Patients were screened based on the above inclusion and exclusion criteria to determine the final samples included in the analysis. In the baseline characteristics of the study subjects, continuous variables with non-Gaussian distribution were presented as median (interquartile range) and compared using the Mann–Whitney U test (for non-normal distribution). Categorical variables were presented as counts (percentages) and compared using the chi-squared test. Statistical significance was set at p < 0.05.

Before analysis, QC-RLSC (quality control-based robust LOESS signal correction) was used to correct the levels of metabolites detected in different batches to minimize errors caused by batch-to-batch variation [31]. After correction, the data were normalized using the Pareto scaling method [32], which includes mean normalization and square root variance transformation (Additional file 1: Figure S3). After the above corrections and standardizations, the data were ready for further analysis.

### Data analysis for metabolomics

Logistic regression and correlation analyses were performed using built-in functions in R (version 4.2.2). The basic function ‘‘glm’’ in R is used to perform univariate and multivariate logistic regression. For the differentiation of metabolomics between CAD patients without diabetes and those with diabetes, univariate logistic regression was employed. To control for potential confounders, traditional clinical risk factors such as age, alanine aminotransferase (ALT), aspartate aminotransferase (AST), body mass index (BMI), estimated glomerular filtration rate (eGFR), hypertension, hyperlipidemia, and gender were incorporated into the multivariate logistic regression. Metabolites with a Benjamini–Hochberg False Discovery Rate (FDR) adjusted p value less than 0.05 were subjected to Kyoto Encyclopedia of Genes and Genomes (KEGG) enrichment analysis using the MetaboAnalyst online tool (https://www.metaboanalyst.ca). In the multivariate logistic regression results, metabolites with FDR < 0.05 in the discovery cohort and P < 0.05 in the validation cohort were defined as characteristic metabolites (CMs). After excluding a single metabolite with a contrasting OR direction, only 22 CMs were remained. The correlation of these metabolites with HbA1c and fasting glucose concentrations was determined using the built-in R function (“cor”). For the development of a diagnostic model for classifying endpoint events of all-cause mortality in coronary heart disease, Least Absolute Shrinkage and Selection Operator (LASSO) [33] selection analysis was applied to clinical variables and characteristic metabolite variables. The optimal value of the adjustment parameter λ was determined by fivefold cross-validation (200 iterations). The selected biomarkers were then used as covariates to construct a binary logistic regression model. The predictive ability and effectiveness of the model were assessed using the area under the curve (AUC) of the receiver operating characteristic (ROC). The Delong test was then used to compare the AUC values of different models. Visualization of major metabolites was facilitated by the R packages “forestplot” and “pheatmap”.

### Reagents and cells

HepaRG cells were obtained from our research group. Pipecolinic acid (CID: 849), L-pipecolic acid (CID: 439227), and 5-oxoproline (CID: 7405) were purchased from Shanghai Macklin Biochemical Technology Co., Ltd. Dulbecco's modified eagle medium (DMEM) culture medium and fetal bovine serum (FBS) were provided by Gibco. The 1% antibiotics (100 × streptomycin-penicillin) were purchased from Procell, and Trizol RNA extraction reagent was purchased from Axygen Biosciences (AG). In addition, the glucose and lactate assay kits were provided by Nanjing Jiancheng Tech Co., Ltd. and the ATP assay kit and bovine serum albumin (BSA) reagent kit were provided by Shanghai Beyotime Biotechnology.

### Preparation of metabolite stock solution

For metabolite intervention, metabolites were dissolved in complete DMEM culture medium (containing 10% FBS and 1% antibiotics) to formulate a 75 mM stock solution. This solution was stored at  − 20 °C and used within one week. During the experiment, the stock solution was diluted to the desired concentrations (50 μM, 250 μM, 500 μM).

### Assay for glucose concentration

5E4 HepaRG cells were seeded into each well of a 12-well plate. After 24 h of attachment, the supernatant was discarded and the cells were rinsed three times with phosphate-buffered saline (PBS). Culture medium containing different drug concentrations (50 μM, 250 μM, 500 μM) was then added to each well. No drug treatment was given in the negative control group. A blank well without cells was also prepared to which standard complete DMEM culture medium was added. After 60 h of drug treatment, 500 μM supernatant was collected and centrifuged at 1000 rpm for 10 min. Glucose concentration was then determined according to the guidelines of the Jiancheng Glucose Assay Kit. Glucose Consumption = Glucose Concentration in Complete Medium—Glucose Concentration in Supernatant after 60 h. Glucose consumption was expressed as the ratio of glucose consumption in each intervention group to the negative control group.

### Measurement of lactate levels

Under metabolite intervention conditions, a concentration of 50 μM was chosen. After 60 h of intervention, 200 μM trypsin was added to each well for 2 min of digestion. Once the cells were completely detached, 200 μM complete culture medium was added to stop the digestion and the samples were collected in 1.5 mL centrifuge tubes. The samples were then centrifuged at 1000 rpm for 5 min, the supernatant was gently discarded, and 200 μL PBS was added to each tube for resuspension. Cells were lysed using an ultrasonic cell disruptor. The supernatant was then collected, and lactate levels were determined using the Jiancheng lactate assay kit according to its instructions.

### ATP assay

10E4 HepaRG cells were seeded in each well of a 6-well plate. The metabolite was adjusted to a concentration of 50 μM. After treatment, 200 μL ATP lysis buffer was added to each well and the cells were lysed on ice for 5 min. The lysate was then centrifuged at 12,000 g for 5 min. A portion of the supernatant was collected and mixed with the ATP detection working solution in a 96-well plate. The concentrations of the calibration curve were determined. After a minimum reaction time of 3 s, chemiluminescence was detected. The results were expressed as the ratio of ATP content in each intervention group to the negative control group.

### Bovine serum albumin (BSA) assay

Similarly, 10E4 HepaRG cells were seeded in each well of a 6-well plate. Each metabolite was added at a concentration of 50 µM. After treatment, 200 μL lysis buffer was added to each well and the cells were lysed on ice for 5 min. The lysate was then centrifuged at 13,000 rpm for 18 min. A portion of the supernatant was removed and mixed with the BSA detection working solution in a 96-well plate. The concentrations of the calibration curve were determined. After a minimum reaction time of 3 s, the absorbance at 540 nm was measured.

### Statistical analysis

Each sample within the same batch of data was measured at least twice and the mean was calculated. For biological replicates, the experiment was independently repeated at least three times. One-way analysis of variance was used to test for statistical differences in GraphPad Prism 9, with a P value of less than 0.05 considered significant.

### RNA sequence assay and transcriptomics analysis

10E4 HepaRG cells were seeded in each well of a 6-well plate. After 24 h of attachment, cells were treated with regular complete culture medium, 50 µM pipecolic acid, and 50 µM L-pipecolic acid complete culture medium for 60 h. Total RNA was extracted using the Trizol reagent kit according to the manufacturer's instructions. RNA quality was evaluated and verified. After total RNA extraction, eukaryotic mRNA was enriched. The enriched mRNA was then fragmented and reverse transcribed to cDNA. The purified double-stranded cDNA fragments underwent several processes before sequencing by Gene Denovo Biotechnology Co. on the Illumina Novaseq6000 platform. Post-filtering of clean reads, the data was aligned first with Ribosome RNA (rRNA) and then with the reference genome. To ensure the precision of subsequent analyses, corrections were made for gene length or transcript length, followed by a correction for sequencing depth. After obtaining Transcripts Per Million (TPM) values for genes, further analyses were performed. Principal component analysis (PCA) was performed using the R package ‘‘gmodels’’. Differential expression analysis of RNAs was performed using ‘‘DESeq2’’. Transcripts with a p-value below 0.05 and an absolute fold change of ≥ 1.3 were classified as differentially expressed genes/transcripts. The temporal expression profile of these genes was then examined, followed by KEGG enrichment analyses for each profile. All of the above statistical analyses were performed using the OmicShare tools.

## Results

### Study workflow overview

**Fig. 1:**
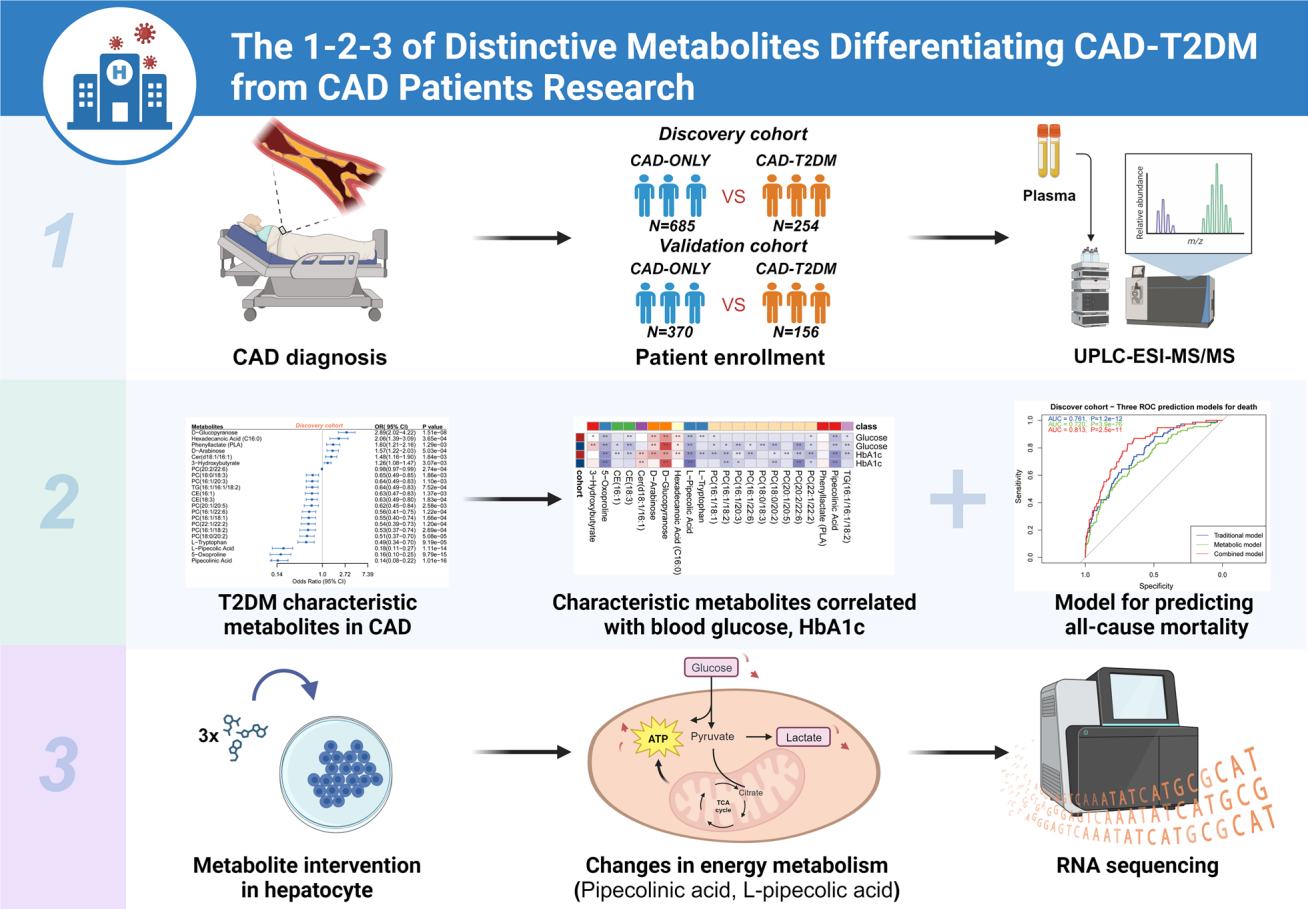
Study design There were 3 steps to the design of this study. Phase 1: Patients with CAD, a total of 1465 individuals from three centers, were stratified into discovery and validation groups based on different enrollment times. widely metabolomics was used to detect plasma metabolites and lipids. Phase 2: Using multivariate logistic regression, 22 characteristic metabolites (CMs) were identified that were associated with CAD-T2TM (vs. CAD-ONLY). Relationships between these CMs and fasting blood glucose, HbA1c and all-cause mortality were established. Phase 3: At the cellular level, three CMs significantly negatively correlated with T2DM were validated. Only pipecolic acid and L-pipecolic acid induced changes in cellular energy metabolism. Finally, transcriptional data provided insight into potential targets and pathways affected by these two metabolites

The study workflow is shown in Fig. 1, created with BioRender.com.

### Participant profile overview

In this study, we enrolled a total of 1465 patients diagnosed with CAD from three different medical centers in China. The discovery cohort, which originated from the Guangdong Provincial People’s Hospital, included 685 patients with CAD and an additional subset of 254 patients who manifested a concomitant presence of CAD and T2DM (Table 1). Compared to the CAD-ONLY group, the CAD-T2DM group had a slightly older mean age and a female predominance. Hypertension was more common in the CAD-T2DM group, while the incidence of hyperlipidemia was similar in both groups. Biochemical markers such as triglyceride (TG), fasting blood glucose (GLUC) and hemoglobin A1c (HbA1c) were significantly higher in the CAD-T2DM group (P < 0.05). The baseline information for the validation cohort of 526 patients is presented in Additional file 2 Table S3.

**Table 1:** Clinical baseline data of patients in the discovery cohort

	CAD-ONLY	CAD-T2DM	*P value*
	n = 685	n = 254	
Demographic data			
Age (years)	62.60 (56.00,70.20)	64.40 (57.55,71.85)	0.030
Sex (male), %	83.50	72.05	< 0.001
BMI (kg/m^2^)	24.00 (22.00,26.00)	24.50 (22.00,27.00)	0.011
Comorbidities			
Hypertension, %	57.08	65.75	0.016
Hyperlipidemia, %	10.51	12.60	0.365
Biochemical measurements			
ALT, U/L	24.00 (18,34)	24.00 (18,34)	0.633
AST, U/L	24.00 (20,30)	23.00 (19,30)	0.065
eGFR, ml/min/1.73 m^2^	88.81 (74.24,104.60)	87.53 (69.28,103.51)	0.475
CK, U/L	88.00 (64.00,129.00)	85.00 (57.50,110.00)	0.028
CKMB, U/L	6.25 (4.50,8.73)	7.00 (5.00,9.20)	0.038
CREA, μmol/L	83 (71,96)	82 (69,97)	0.611
CHOL, mmol/L	4.07 (3.49,4.85)	4.18 (3.56,4.91)	0.243
LDLC, mmol/L	2.44 (1.88,3.16)	2.46 (1.91,3.03)	0.777
HDLC, mmol/L	0.92 (0.79,1.10)	0.955 (0.79,1.09)	0.799
APOA, g/L	1.00 (0.86,1.17)	1.01 (0.86,1.20)	0.579
TG, mmol/L	1.30 (0.97,1.78)	1.48 (1.11,2.11)	< 0.001
Lp(a), mg/L	179.40 (86.74,428.64)	178.09 (79.86,361.14)	0.333
proBNP, pg/mL	197.10 (63.19,672.28)	254.70 (85.75,878.00)	0.082
GLUC, mmol/L	5.45 (4.90,6.22)	7.90 (6.33,10.84)	< 0.001
HbA1c, %	6.00 (5.70,6.30)	7.40 (6.60,8.55)	< 0.001

Data are shown as median (IQR) or n (%). Chi-square (χ2) tests were used for categorical variables. T-tests or Mann–Whitney U tests were used for normally and non-normally distributed variables, respectively. *BMI *body mass index, *ALT* alanine aminotransferase, *AST* aspartate aminotransferase, *eGFR* estimated glomerular filtration rate, *CK* creatine kinase, *CKMB* MB isoenzyme of creatine kinase, *CREA* Creatinine, *CHOL* cholesterol, *LDLC* low-density lipoprotein cholesterol, *HDLC* high-density lipoprotein cholesterol, *APOA* apolipoprotein, *TG* triacylglycerol, Lp(a): lipoprotein(a), *proBNP *N-terminal pro-B-type natriuretic peptide, *GLUC* fasting blood glucose, *HbA1c* hemoglobin A1c

### Metabolomic landscape in CAD-T2DM patients

To delineate the intricate relationship between specific metabolites and CAD-T2DM, initial univariate logistic regression analyses were conducted on an array of 202 annotated metabolites and 667 lipidomic entities. Subsequent to FDR adjustment, a total of 192 metabolites remained significantly correlated with T2DM (FDR < 0.05) (Additional file 2: Table S4). The validation cohort corroborated the significance of 95 metabolites (P < 0.05) (Additional file 2: Table S5). Further multivariate logistic regression analyses, adjusted for variables such as age, ALT, AST, BMI, eGFR, gender, hypertension and hyperlipidemia, revealed that 65 metabolites, including pipecolinic acid (OR: 0.14 [0.08–0.22]), retained their statistical significance (FDR < 0.05) (Additional file 2: Table S6). These metabolites were predominantly implicated in the biosynthetic pathways of phenylalanine, tyrosine, and tryptophan, as well as glycerophospholipid metabolism (Additional file 1: Figure S4).

**Fig. 2:**
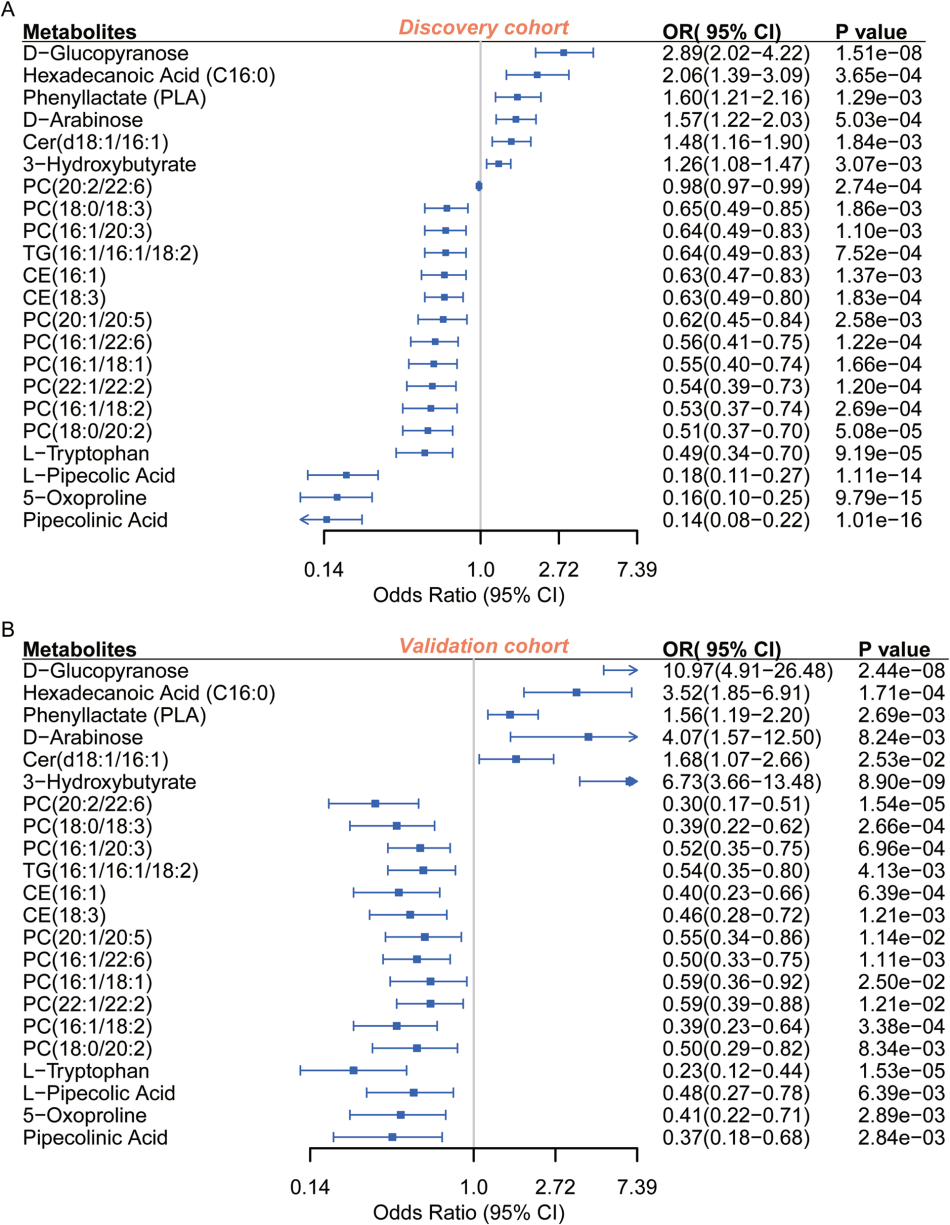
Forest plot of characteristic plasma metabolites associated with T2DM risk in CAD patients. (Discovery cohort FDR < 0.05, Validation cohort P < 0.05). Multivariate logistic regression in the discovery **A** and validation **B** cohorts, adjusted for age, ALT, AST, BMI, eGFR, hypertension, hyperlipidemia, and gender

The independent validation cohort was utilized to verify the metabolites identified in our study (Additional file 2: Table S7). Then, a confluence of 23 candidate characteristic metabolites was discerned (Additional file 2: Table S8), intersecting between the 65 metabolites that retained statistical significance following multivariate adjustment and those substantiated by the validation cohort (P < 0.05). Upon the exclusion of 3-indolebutyric acid, which demonstrated an inverse Odds Ratio (OR) (OR in discovery cohort: 0.56 [0.41–0.75]; OR in validation cohort: 2.74 [1.4–5.49]), a total of 22 characteristic metabolites (hereafter called CMs.) were retained as salient biomarkers intricately associated with T2DM risk (Additional file 2: Table S9). Among these, 16 metabolites were found to be negative correlated with T2DM susceptibility, while 6 metabolites were positively correlated. The distribution of the normalized concentrations of the CMs in the two groups is as follows (Additional file 1: Figures S5, S6). Notably, the monosaccharides D-glucopyranose (OR: 2.89 [2.02–4.22]) and D-arabinose (OR: 1.57 [1.22–2.03]) were implicated in augmenting the risk of T2DM in the discovery cohort. Hexadecanoic acid was identified as a significant risk factor for T2DM (OR: 2.06 [1.39–3.09]), second only to D-glucopyranose. Phenyllactate, a metabolic byproduct of phenylalanine catabolism, was conspicuously elevated in patients diagnosed with phenylketonuria. Ceramide (Cer) (d18:1/16:1) emerged as the sole lipid moiety positively correlated with T2DM risk (OR: 1.48 [1.16–1.90]). In our results, pipecolinic acid and L-pipecolic acid (OR: 0.18 [0.11–0.27]) were identified as the most important potential protective factors against T2DM. Moreover, our investigation unveiled an array of Phosphatidylcholines (PCs) and Cholesteryl Esters (CEs), suggesting their putative protective role in the pathogenesis of T2DM (Fig. 2).

### Correlation of characteristic metabolites with GLUC and HbA1c

**Fig. 3:**
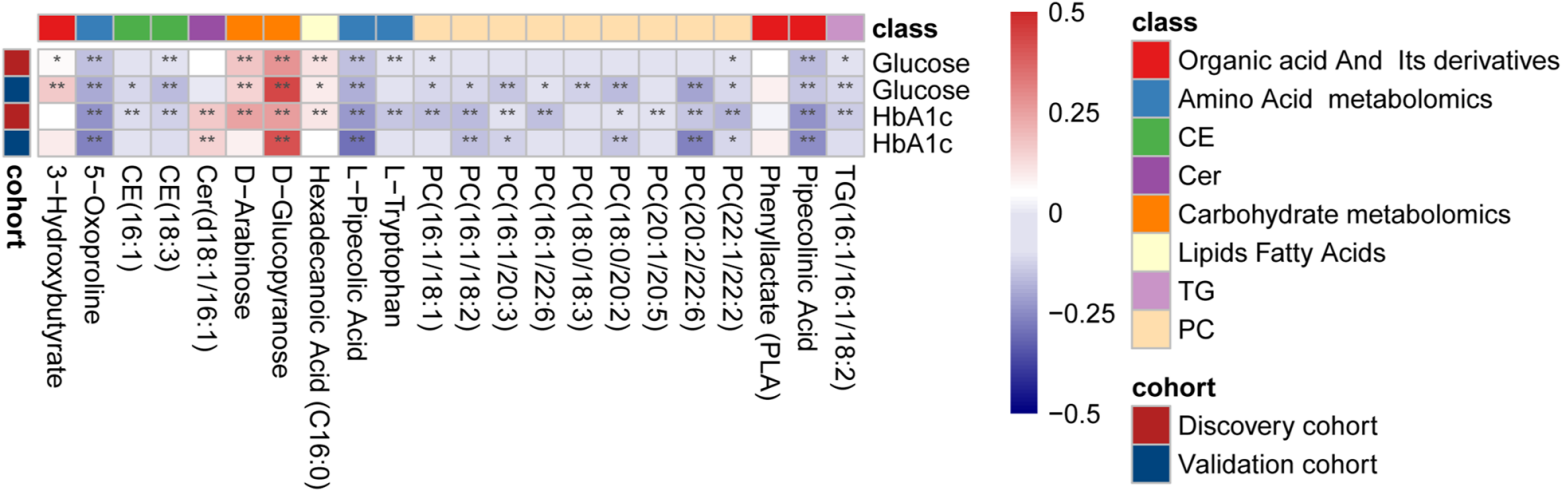
Spearman correlation heatmap of characteristic metabolites with GLUC and HbA1c levels in CAD patients. The intensity of the color in the heatmap represents the strength of the correlation, with deeper colors indicating stronger associations. An asterisk (*) indicates statistical significance at P < 0.05

Fasting blood glucose and glycated hemoglobin are established clinical biomarkers of T2DM that reflect fluctuations in blood glucose concentrations over time. To determine whether the CMs of T2DM are directly associated with changes in body glucose levels, we first examined the correlation of 22 metabolites with GLUC and HbA1c (Fig. 3, Additional file 2: Tables S10, S11).

For GLUC, both the discovery and validation cohorts showed that D-glucopyranose and D-arabinose were significantly positively correlated with GLUC, whereas pipecolinic acid, L-pipecolic acid, 5-oxoproline, TG(16:1/16:1/18:2), CE(18:3), and PC(18:0/20:2) were all significantly negatively correlated with GLUC. For HbA1c in both cohorts, D-glucopyranose and Cer(d18:1/16:1) were significantly positively correlated with HbA1c, whereas pipecolinic acid, 5-oxoproline, l-pipecolic acid, PC(16:1/18:2), PC(22:1/22:2), PC(18:0/20:2), PC(16:1/20:3), and CE(18:3) were all significantly negatively correlated with HbA1c. Although these metabolites were correlated with T2DM in logistic regression, they were not correlated with GLUC or HbA1c, suggesting their potential role as stage markers not directly related to blood glucose levels.

### Pipecolinic acid and L-pipecolic acid promote ATP production in hepatocytes

**Fig. 4:**
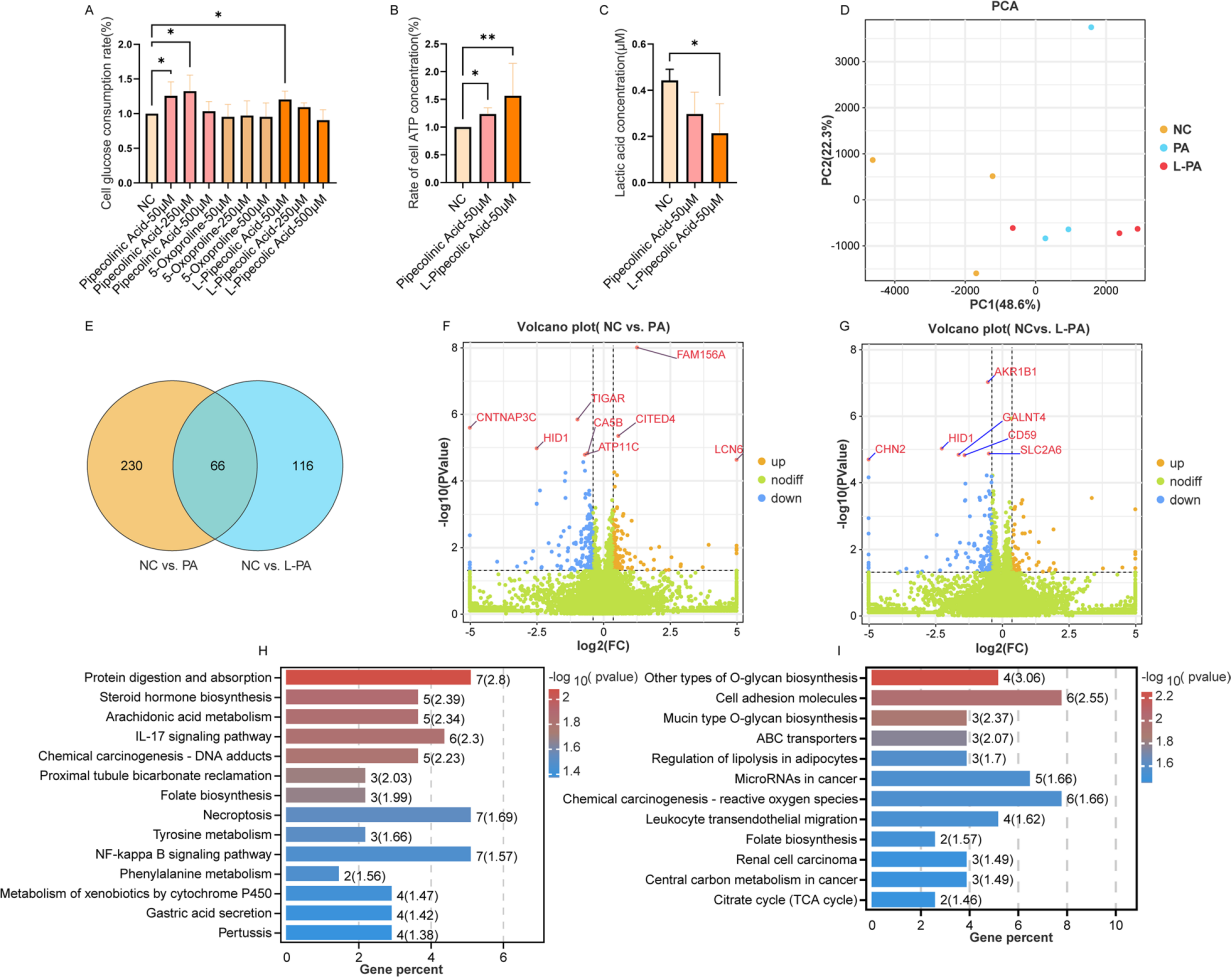
Effects of PA and L-PA on glucose metabolism and transcriptional changes in hepatocytes. **A** Glucose consumption in HepaRG cells influenced by metabolites at different concentrations over a period of 60 h. **B** Intracellular ATP levels and **C** intracellular lactate levels under the same conditions. Multiple group comparisons were performed using one-way analysis of variance (ANOVA). Significance is indicated by * for P < 0.05 and ** for P < 0.01. **D** Principal component analysis (PCA) visually depicts the differences between groups after PA and L-PA intervention. **E** Venn diagram shows the overlap of differentially expressed genes (DEGs) between the comparison groups (NC vs. PA) and (NC vs. L-PA). **F**, **G** Volcano plots show the DEGs for the comparison groups (NC vs. PA) and (NC vs. L-PA), respectively, with DEGs with FDR < 0.05 labeled. **H**, **I** KEGG pathway enrichment analysis of DEGs from the comparison groups (NC vs. PA) and (NC vs. L-PA). The x-axis indicates the percentage of DEGs relative to the total genes in the pathway (expressed in %), with numbers in parentheses representing -log10 (p-value)

To further elucidate whether the CMs influence glucose consumption and utilization, we validated three metabolites that were significantly negatively correlated with diabetes. After treating HepaRG cell with different concentrations (50 μM, 250 μM, 500 μM) of pipecolinic acid, 5-oxoproline, and L-pipecolic acid for 60 h, the 50 μM concentration of pipecolinic acid and Lpipecolic acid (In the following, PA and L-PA refer specifically to the groups treated with 50 µM pipecolinic acid and 50 µM L-pipecolic acid, respectively.) significantly increased cellular glucose consumption (Fig. 4A). Both significantly increased ATP levels (Fig. 4B). BSA protein levels showed no difference, ruling out the influence of cell proliferation (Additional file 1: Figure S7). Changes in intracellular lactate levels indicated that pipecolinic acid and L-pipecolic acid convert excess glucose to ATP through oxidative phosphorylation (Fig. 4C).

### RNA Sequencing identifies genes driven by different isomers of pipecolic acid

To uncover potential targets influenced by pipecolinic acid and L-pipecolic acid, we used high-throughput RNA Sequencing (RNA-Seq) to identify differences at the transcriptional level. In Principal Component 1 (PC1), the two drug treatment groups and the negative control (NC) group exhibited a clear separation, indicating significant differences in gene expression. The PA and L-PA showed some degree of separation in Principal Component 2 (PC2) (Fig. 4D). The results of differential gene expression analysis with P < 0.05 after PA and L-PA treatment are listed in Additional file 2: Tables S12, S13, respectively.

Using a threshold of P < 0.05 and a fold change greater than 1.3, we identified 296 and 182 differentially expressed genes (DEGs) in the two comparison groups (NC vs. PA and NC vs. L-PA), respectively (Fig. 4D). 66 DEGs were common to both groups (Fig. 4E), and both sets of DEGs showed similar expression patterns (Additional file 1: Figure S8). Volcano plots for the DEGs of the two comparison groups are shown in Fig. 4F, G, with genes with FDR less than 0.05 labeled. The common differential gene HID1 (Fig. 4F) was significantly downregulated in both groups. Previous RNA-Seq transcriptomic studies found that HID1 mRNA levels were significantly correlated with obesity and glucose metabolic parameters in human subcutaneous and adipose tissues after Bonferroni correction for multiple testing [34]. After PA treatment, the transcript levels of the glycolysis inhibitor TIGAR [35] and the glucose neogenesis-related gene CA5B were downregulated, while the transcript levels of LCN6, FAM156A and CITED4 were upregulated. In the comparison group (NC vs. L-PA), ARID5B was an upregulated differential gene, while transcript levels of AKR1B1, SLC2A6, and GALNT4 all decreased.

To further comprehend the biological context of these differentially expressed genes, KEGG pathway enrichment analysis was executed. Treatment with PA and L-PA revealed distinct as well as overlapping pathway enrichment profiles, contributing to a deeper understanding of the molecular mechanisms underlying Pipecolic Acid's impact on gene expression and metabolic regulation. In the PA comparison group (NC vs. PA), 242 pathways were identified (Additional file 2: Table S14). Beyond the lipid and carbohydrate metabolism pathways (Fig. 4H), significant enrichment was observed in the IL-17 and NF-kappa B signaling pathways. In addition, the L-PA comparison group (NC vs. L-PA) exhibited enrichment in 206 pathways (Additional file 2: Table S15). These pathways showed notable associations with glucose and lipid metabolism-related pathways, including other types of O-glycan biosynthesis, mucin-type O-glycan biosynthesis, folate biosynthesis, citrate cycle (TCA cycle), and regulation of lipolysis in adipocytes (Fig. 4I). Notably, no pathways related to glycation modifications were observed in the PA comparison group (NC vs. PA).

### Characteristic metabolites enhance predictive accuracy for clinical endpoints in cardiovascular diseases

**Fig. 5:**
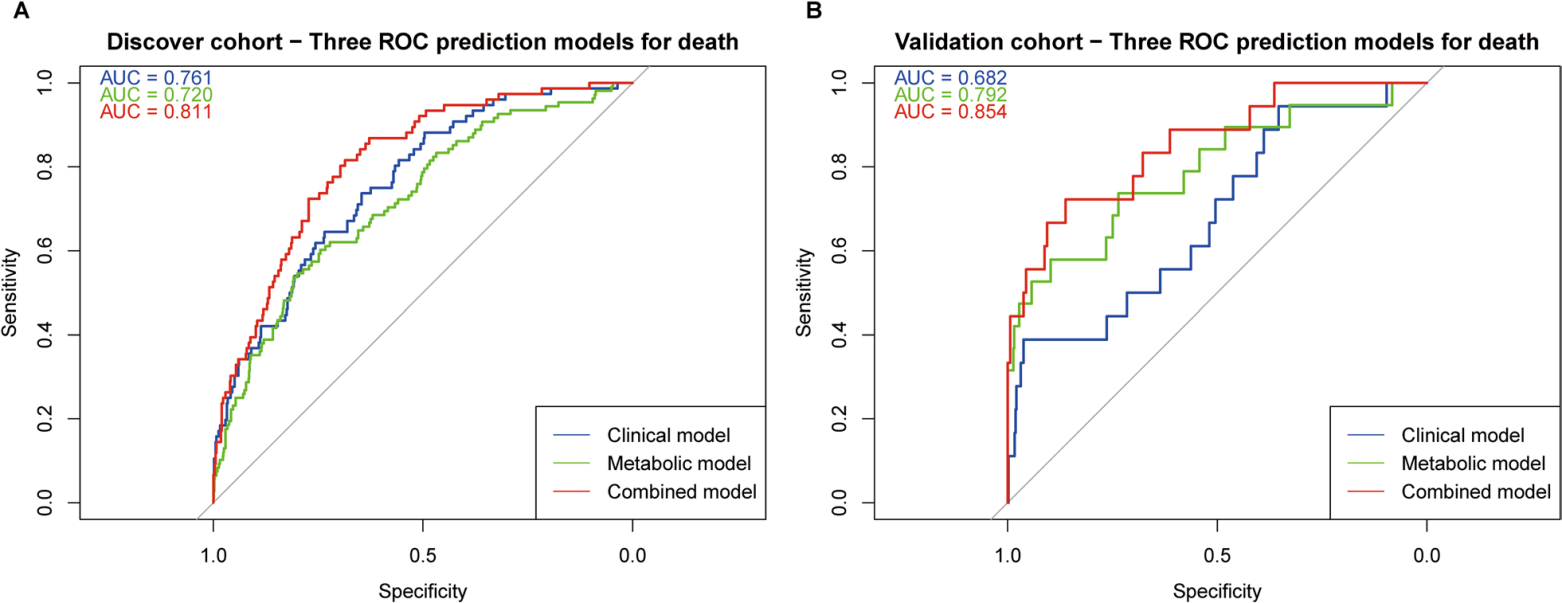
Models for predicting mortality in CAD patients based on characteristic metabolites. **A** For the discovery cohort and **B** For the validation cohort. Clinical model: The clinical variables selected by LASSO regression are age, AST, CREA, proBNP, and gender. Metabolic model: The metabolites selected by LASSO regression are 3-hydroxybutyrate, CE(16:1), CE(18:3), Cer(d18:1/16:1), D-arabinose, D-glucopyranose, hexadecanoic acid(C16: 0), L-pipecolic acid, PC(16:1/18:1), PC(16:1/20:3), PC(18:0/18:3), PC(18:0/20:2), PC(20:1/20:5), PC(22:1/22:2), phenyllactate, pipecolinic acid, and TG(16:1/16:1/18:2). Combined Model: This model integrates both clinical and metabolite variables for prediction

We focused on the association between the 22 identified CMs and CAD all-cause mortality. For this analysis, clinical endpoints were used as predictive indicators. Using LASSO, we screened traditional clinical variables and metabolomic variables separately (fivefold cross-validation, 200 replicates, frequency of occurrence > 100 times, Additional file 2: Table S16) to identify the most important features. We then compared the predictive power of the clinical model, the metabolic model, and the combined model. In the discovery cohort, the combined model (AUC = 0.813, P value (vs. Clinical model): 0.001) had a higher predictive power than both the clinical model (AUC = 0.761) and the metabolic model (AUC = 0.720) (Fig. 5A). This result was also confirmed in the validation cohort (Fig. 5B). Differences between the models were determined using the Delong test (Additional file 2: Table S17).

## Discussion

In this study, we have investigated the metabolomic profile of plasma sample from CAD as well as patients with CAD and T2DM. Statistical analysis of the metabolomic data revealed 192 and 95 significantly altered metabolic signatures of CAD-T2DM patients in discovery (FDR < 0.05) and validation cohort (P < 0.05), respectively. Further multivariate logistic regression analyses, adjusted for variables revealed that 65 metabolites retained their statistical significance in discovery cohort. After intersection of 65 metabolites in the validation cohort and the discovery cohort, 23 candidate CMs were identified. Upon the exclusion of 3-indolebutyric acid, which demonstrated an inverse OR, a total of 22 CMs were retained as salient biomarkers intricately associated with T2DM risk. Among these, 16 metabolites exhibited negative correlation with T2DM susceptibility, while 6 metabolites were positively correlated. The positive metabolites including D-glucopyranose, hexadecanoic acid, PLA, D-arabinose, Cer(d18:1/16:1) and 3-hydroxybutyrate were implicated in augmenting the risk of T2DM in the two cohorts.

We observed the exclusive accumulation of D-glucopyranose and D-arabinose in CAD-T2DM patients. D-glucopyranose represents the natural form of glucose, while D-arabinose, a rare pentose, serves as a carbon source for bacteria [36]. Previous studies suggest a marked growth inhibitory effect of D-arabinose on the nematode Caenorhabditis elegans, possibly due to disruption of D-ribose and D-fructose metabolism [37]. Regarding PLA, it is significantly elevated in patients with phenylketonuria, and PLA levels show a positive correlation with T2DM in our study. However, in a cohort study of 134 individuals (healthy and T2DM), PLA levels decreased significantly in the T2DM group. This suggests that compared to individuals with T2DM alone (vs. healthy individuals) [38], our CAD-T2DM may have different PLA metabolite characteristics than CAD-ONLY. In addition, there is evidence that treatment activates PPAR-γ2, which promotes adipocyte differentiation and glucose uptake [39]. Further studies are needed to gain a deeper understanding of these differences. 3-Hydroxybutyrate, originating from the increased β-oxidation of free fatty acids [40], is found in higher concentrations in the plasma of T2DM patients [41] and has been shown to ameliorate insulin resistance in T2DM mice through the HCAR2/Ca^2+^/cAMP/PKA/Raf1/ERK1/2/PPARγ pathway [42].

Pipecolic acid, a metabolite of lysine found in human physiological fluids such as urine, plasma and CSF, is an important regulator of immunity in plants and humans alike [43]. Normal adults excrete pipecolic acid primarily as the D-enantiomer even though it is present in the blood stream mainly as the L-enantiomer. Despite some studies reporting a significant increase in D-pipecolic acid, L-pipecolic acid concentrations in the Human Diabetic Corneal Stroma compared to healthy individuals [44], other research [45] revealed that 81 metabolites were significantly changed before T2DM onset, and pipecolinic acid was reported to be negatively significantly associated with future T2DM risk. Previous studies have reported a reduction in pipecolic acid levels in individuals with T2DM [46]. Similarly, our study shows that pipecolinic acid and L-pipecolic acid have a protective effect in CAD-T2DM. Moreover, our results innovatively revealed at the cellular and molecular level that pipecolinic acid and L-pipecolic acid both convert excess glucose to ATP for energy through oxidative phosphorylation in hepatocytes.

Elevated levels of tryptophan are often accompanied by an increased risk of T2DM [47]. However, in patients with CAD, elevated Tryptophan levels are associated with a reduced mortality rate from coronary artery diseases [48]. Metabolites involved in glutathione metabolism, including 5-oxoproline, have been previously identified as markers for diabetes [49]. The first longitudinal analysis conducted in SOALS suggests that 5-oxoproline is associated with a 50% higher risk of T2DM [50]. The alteration in amino acid metabolism may impact the disease mechanism in CAD patients with T2DM [49]. This change implies that some biomarkers effective in healthy populations may not be suitable for patients with comorbid conditions.

Furthermore, to the best of our knowledge, this is the first metabolomic comparison including lipidomics between CAD-ONLY and CAD-T2DM. The presence of a large number of lipids among the CMs indicates a concomitant alteration in lipid metabolism. A portion of phosphatidylcholine (PC), cholesteryl ester (CE), and triglycerides (TG) are negatively correlated with T2DM, whereas hexadecanoic acid(C16:0) and Cer(d18:1/16:1) are positively correlated with T2DM. The elevated levels of hexadecanoic acid in individuals with diabetes may can be explained by an increase in harmful complex lipid synthesis, impairment of cellular organelle function, and promotion of receptor-mediated inflammation [51–54]. In simple terms, intracellular levels of palmitic acid rise above the mitochondrial oxidation limit and are converted into harmful complex fatty acid-derived lipids such as diacylglycerol and Cer. PC, as one of the most abundant phospholipids in mitochondria, plays a critical role in maintaining mitochondrial function [55]. Excessive lipid supply and reduced mitochondrial oxidative capacity can impair β-oxidation, leading to the accumulation of Cer [56].

In our investigation, comprehensive high-throughput RNA-Seq analysis identified 296 and 182 differentially expressed genes in the two comparison groups (NC vs. PA and NC vs. L-PA), respectively, with 66 DEGs being common to both groups and showing similar trends. For example, TIGAR, an intrinsic inhibitor of glycolysis [35, 57], and ARK1B1, a known activator of the polyol pathway often associated with long-term diabetic complications [58], as well as the glucose neogenesis-related gene CA5B [59, 60], were all significantly decreased after PA and L-PA intervention. Conversely, transcript levels of LCN6, which enhances skeletal muscle mitochondrial function [61, 62], increased significantly after intervention. KEGG enrichment analysis revealed a primary clustering of alterations in glucose and lipid metabolism and inflammatory pathways for both comparison groups (NC vs. PA and NC vs. L-PA). Folate biosynthesis emerged as a commonly enriched pathway, where chronic folate deficiency leads to disruptions in glucose and lipid metabolism and subsequent cognitive dysfunction in mice [63]. In addition, DEGs in L-PA affected the O-glycan biosynthesis and TCA cycle pathways, which may explain the increased ATP generation observed in the L-PA group compared to the PA group. In contrast, DEGs from the NC vs. PA group showed significant enrichment in the IL-17 and NF-kappa B pathways [64, 65]. In conclusion, both pipecolinic acid and L-pipecolic acid are capable of modifying hepatic glucose utilization, with the transcriptomic data shedding light on the potential target sites and pathway differences for the metabolic actions of these two metabolites, including their impact on ATP production.

Given the enrichment of DEGs in NC vs. PA and NC vs. L-PA in diverse pathways, including those related to immune and signaling activation, and acknowledging the well-documented influence of diabetes on cardiovascular disease progression. There have been many studies on the relationship between diabetes and cardiovascular disease [66–68]. Globally, cardiovascular disease affects approximately 32.2% of all persons with T2DM, and the major cause of death for T2DM patients is cardiovascular disease [67, 68]. Diabetes mellitus is a risk factor for cardiovascular disease and has been associated with 3- to fourfold higher mortality [66]. We conducted LASSO analysis to value the prediction of metabolites on cardiovascular death. the combined model with metabolites is more comprehensive than clinical model and the metabolic model.

Our study has several limitations. First, because all the patients in this study came from a cohort of CAD patients without healthy individuals, the characteristics examined in this article are based only on CAD patients, and the difference in the metabolic spectrum between CAD patients and healthy individuals could not be determined. Secondly, the differential timeline for enrollment (2010–2014 in discovery vs. 2017–2018 for validation) and the low participant numbers in the Sun Yat-sen University and the Xiangya Hospital of Central South University may introduce confounding for center-specific features. Third, we only recorded the presence of T2DM at the time of enrollment and did not follow up the incidence of new-onset diabetes, thereby losing information on the time variable. Fourth, the lack of information on the use of hypoglycemic agents prevents correction for potential bias introduced by such medications. Fifth, more than 80% of the patients in this cohort are male. Although we corrected for this covariate during the analysis, our results may still have some gender bias. Sixth, the metabolomic data in this study were not collected at one time, and with the iterative updating of technology, the range of detectable metabolites is different. Therefore, we only included repeatedly appearing metabolites, which undoubtedly lost some metabolite information. In addition, multiple testing can lead to batch effects, which may still influence the results despite correction. Seventh, A triple quadrupole low resolution mass spectrometer with sMRM mode was used to establish the database information of the three samples of pipecolic acid (pipecolinic acid, D-pipecolic acid, and L-pipecolic acid). We only verified the effect of pipecolinic acid and L-pipecolic acid on glucose metabolism, and the role of D-pipecolic acid was neglected in this study, so the role of D-pipecolic acid in metabolism needs to be further investigated. Finally, the direct addition of metabolites to cells without validation in a disease model may not fully elucidate the role of these metabolites in disease contexts. Future studies using a more comprehensive approach, including the use of disease models and a diverse patient cohort, are essential to validate and extend our findings.

## Conclusion

This study is based on a cohort of 1465 CAD patients from three centers. Plasma metabolic characteristics were differentiated between CAD patients without T2DM and those with concomitant T2DM through comprehensive, widely targeted metabolomics and lipidomics profiling analyses. We identified potential metabolic biomarkers and constructed a powerful model using characteristic metabolites to predict all-cause mortality endpoints. Moreover, we explored the potential functional pathways of characteristic metabolites through transcriptomic sequencing and cellular experiments. Intriguingly, pipecolic acid and L-pipecolic acid, the key metabolites impact on ATP production, may act as the intervention points to improve energy metabolism of CAD-T2DM patients. In conclusion, our study provides novel insights into the further mechanism research, prophylaxis and treatment of comorbidity of CAD and T2DM.

### Supplementary Information

**Additional file 1: Figure S1.** Flowchart for patient enrolment. **Figure S2.** Mass spectra of quality control (QC) samples**. Figure S3.** Density plots of the data. **Figure S4.** Pathway enrichment analysis of metabolites associated with T2DM (FDR<0.05). **Figure S5.** Distribution of characterized metabolite levels in the discovery cohort. **Figure S6.** Distribution of characterized metabolite levels in the validation cohort. **Figure S7.** BSA content after 60 h metabolite intervention. **Figure S8.** Heatmap of differentially expressed genes (DEGs).

**Additional file 2: Table S1.** The ionization modes and ion pairs of the metabolites. **Table S2.** The ionization modes and ion pairs of the lipid species. **Table S3.** Clinical baseline data of patients in the validation cohort. **Table S4.** One factor logistic regression of diabetes in the discovery cohort. **Table S5.** One factor logistic regression of diabetes in the validation cohort. **Table S6.** Multivariate logistic regression of diabetes in the discovery cohort. **Table S7.** Multivariate logistic regression of diabetes in the validation cohort. **Table S8.** Multivariate logistic results of candidate characteristic metabolites in two cohort. **Table S9.** Multivariate logistic results of characteristic metabolites in two cohorts. **Table S10.** Relationship between plasma glucose and metabolites associated with diabetes. **Table S11.** Relationship between plasma HbA1c and metabolites associated with diabetes. **Table S12.** Difference in transcription levels after PA treatment (P<0.05). **Table S13.** Difference in transcription levels after L-PA treatment (P<0.05). **Table S14.** KEGG pathway analysis of DEGs in NC vs. PA. **Table S15.** KEGG pathway analysis of DEGs in NC vs. L-PA. **Table S16.** Feature inclusion frequency using LASSO based feature selection for death prediction. **Table S17.** Model performance measures (95% CIs) for death prediction.

**Additional file 3:** Transcriptome Data.

## Data Availability

Patient-related data, including profiles, will not be made publicly available for privacy reasons. However, RNA sequencing expression data are available in the Additional file 3 for use by researchers who comply with ethical and legal requirements. For data access or inquiries, please contact the study data administrator or the relevant research institution.

## Abbreviations

CAD: Coronary artery disease
T2DM: Type 2 diabetes mellitus
PCI: Percutaneous coronary intervention
GLUC: Fasting blood glucose
HbA1c: Hemoglobin A1c
CREA: Creatinine
CK: Creatine kinase
EDTA: Ethylenediaminetetraacetic acid
LC–ESI–MS/MS: Liquid chromatography electrospray ionization tandem mass spectrometric
UPLC-MS/MS: Ultraperformance liquid chromatography tandem mass spectrometry
MWDB: Metware database
MRM: Multiple reaction monitoring
QC: Quality control
QC-RLSC: Quality control-based robust LOESS signal correction
ALT: Alanine aminotransferase
AST: Aspartate aminotransferase
BMI: Body mass index
eGFR: Estimated glomerular filtration rate
FDR: False discovery rate
KEGG: Kyoto Encyclopedia of Genes and Genomes
CMs: Characteristic metabolites
LASSO: Least absolute shrinkage and selection operator
AUC: Area under the curve
ROC: Receiver operating characteristic
DMEM: Dulbecco’s modified eagle medium
PBS: Phosphate-buffered saline
FBS: Fetal bovine serum
AG: Axygen biosciences
BSA: Bovine serum albumin
PBS: Phosphate-buffered saline
rRNA: Ribosome RNA
TPM: Transcripts per million
PCA: Principal component analysis
TG: Triglycerides
OR: Odds ratio
Cer: Ceramide
PC: Phosphatidylcholine
CE: Cholesteryl ester
PA: Groups treated with 50 µM pipecolinic acid for 60 h
L-PA: Groups treated with 50 µM L-pipecolic acid for 60 h
PC1: Principal component 1
RNA-Seq: RNA sequencing
NC: Negative control
PC2: Principal component 2
DEGs: Differentially expressed genes
