# Supplementary figures and images for: Characterizing the metabolic divide: distinctive metabolites differentiating CAD-T2DM from CAD patients
Source: Cardiovasc Diabetol. 2024 Jan 6;23:14. doi: 10.1186/s12933-023-02102-0 (PMC10771670; doi:10.1186/s12933-023-02102-0)

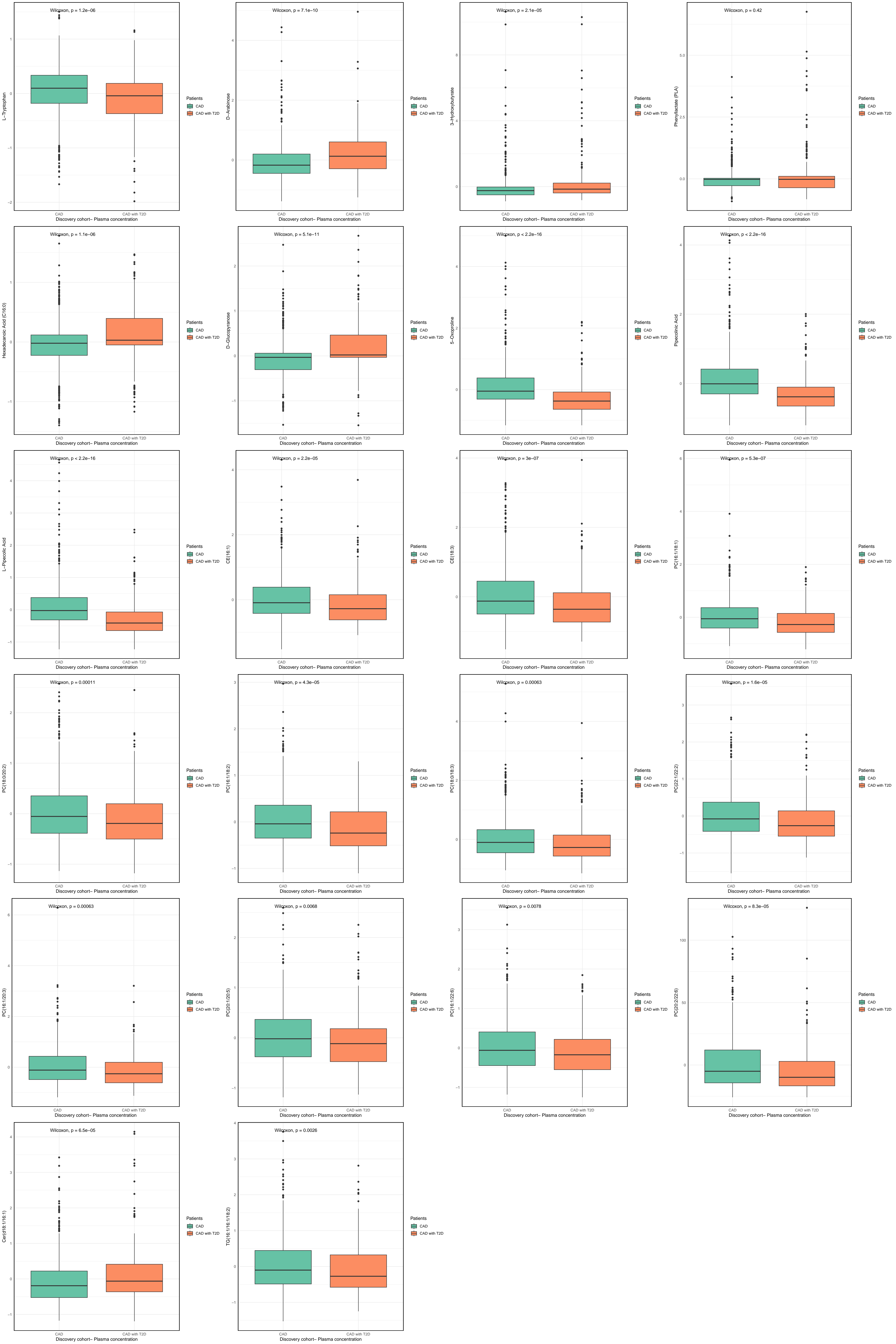

Supplement: Supplementary file 1 — Additional file 1: Figure S1. Flowchart for patient enrolment. Figure S2. Mass spectra of quality control (QC) samples. Figure S3. Density plots of the data. Figure S4. Pathway enrichment analysis of metabolites associated with T2DM (FDR<0.05). Figure S5. Distribution of characterized metabolite levels in the discovery cohort. Figure S6. Distribution of characterized metabolite levels in the validation cohort. Figure S7. BSA content after 60 h metabolite intervention. Figure S8. Heatmap of differentially expressed genes (DEGs). [file 12933_2023_2102_MOESM1_ESM.zip › Supplement figures/Supplementary Figure5.pdf]

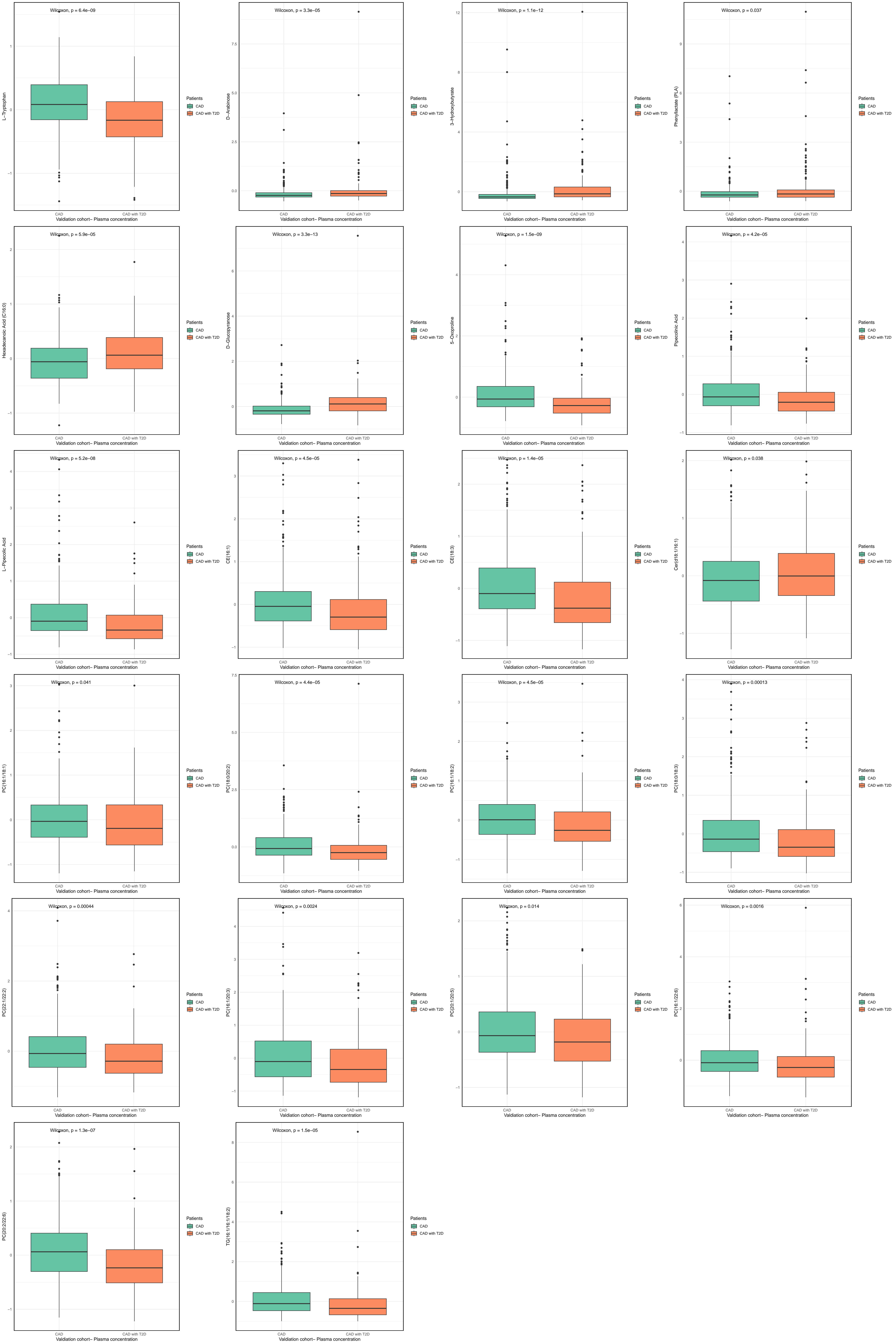

Supplement: Supplementary file 1 — Additional file 1: Figure S1. Flowchart for patient enrolment. Figure S2. Mass spectra of quality control (QC) samples. Figure S3. Density plots of the data. Figure S4. Pathway enrichment analysis of metabolites associated with T2DM (FDR<0.05). Figure S5. Distribution of characterized metabolite levels in the discovery cohort. Figure S6. Distribution of characterized metabolite levels in the validation cohort. Figure S7. BSA content after 60 h metabolite intervention. Figure S8. Heatmap of differentially expressed genes (DEGs). [file 12933_2023_2102_MOESM1_ESM.zip › Supplement figures/Supplementary Figure6.pdf]
